# Validation of Italian students’ self-ratings on the SSIS SEL brief scales

**DOI:** 10.3389/fpsyg.2023.1229653

**Published:** 2023-10-05

**Authors:** Valeria Cavioni, Elisabetta Conte, Ilaria Grazzani, Veronica Ornaghi, Carmel Cefai, Christopher Anthony, Stephen N. Elliott, Alessandro Pepe

**Affiliations:** ^1^Department of Humanities, Literature, Cultural Heritage, Education Sciences, University of Foggia, Foggia, Italy; ^2^“R. Massa” Department of Human Sciences for Education, University of Milano-Bicocca, Milan, Italy; ^3^Department of Psychology, University of Malta, Msida, Malta; ^4^School of Special Education, School Psychology, and Early Childhood Studies, University of Florida, Gainesville, FL, United States; ^5^Sanford School of Social and Family Dynamics, Arizona State University, Tempe, AZ, United States

**Keywords:** social skills improvement system SEL brief scales, social and emotional learning, measurement invariance, gender invariance, children, adolescents

## Abstract

**Introduction:**

Despite increasing interest in measuring social and emotional learning (SEL), there is a lack of European-validated tools for assessing the efficacy of SEL programs. The aim of this study was to validate an Italian version of the social skills improvement system (SSIS) SEL brief scales–student form.

**Methods:**

Participants were 1,175 students (mean age: 11.02 years; SD: 2.42; range: 8–16 years; males: 46.8%) recruited at schools in Northern Italy.

**Statistical analyses and results:**

Initial confirmatory factor analysis encountered a series of challenges, implying non-convergence of the original five-factor measurement model (self-awareness, self-management, social awareness, relationship skills, and responsible decision-making) based on the Collaborative on Academic Social Emotional Learning (CASEL) competency framework established with students in the United States. Further exploratory and confirmatory analyses supported a four-factor model that remained partially invariant across gender groups. The Italian version of the SSIS SEL brief scales was thus shown to be an efficient measurement tool for estimating social and emotional learning in students.

**Discussion:**

We discuss the implications of findings in relation to selecting valid and reliable instruments for assessing children’s and adolescents’ SEL competencies, while considering the culturally-situated nature of the constructs under study.

## 1. Introduction

Children develop a variety of personal, cognitive, social, and emotional skills as they move from early childhood to middle and late childhood and to adolescence (Cavioni et al., 2021; Grazzani et al., 2022; Martinsone et al., 2022). Assessing these competencies represents a challenge for practitioners, researchers, and policymakers. To address this issue, over the past decade Elliott and colleagues devised the Social Skills Improvement System-Social and Emotional Learning Edition (SSIS SEL) brief scales, a comprehensive, multi-rater (teacher, parent, and student) assessment, which was developed for use with English and Spanish-speaking students aged 3 to 18 years of age in the United States (Anthony et al., 2020; Elliott et al., 2020). This assessment focuses on social and emotional learning (SEL) as defined by the competency framework advanced by the Collaborative on Academic Social Emotional Learning (2013) and Mahoney et al. (2021). The Collaborative on Academic Social Emotional Learning (CASEL) framework, a prominent conceptual model, has been widely adopted to guide SEL interventions and research (Durlak et al., 2015). Developed as an informed conceptual synthesis, the CASEL framework aims to delineate distinct SEL competency domains, each contributing uniquely to students’ social and emotional development. However, despite its conceptual strength, investigations into the factor structure of measures aligned with the CASEL competency framework have unveiled challenges, particularly regarding the empirical evidence of the framework’s domains. As a way of example, in the context of a large-scale assessment of SEL in various European countries as part of the European PROMEHS Project^1^ on promoting mental health in schools (Cefai et al., 2022; Martinsone et al., 2022; Cavioni et al., 2023; Conte et al., 2023), Anthony et al. (2023) noted that the original CASEL framework underpinning the SSIS SEL encounters critical model fit issues when applied to European data. Findings have highlighted the existence of high overlap among some CASEL SEL competency domains when assessed using empirical techniques like factor analysis. Such empirical difficulties have raised questions about the distinctiveness and empirical fit of the framework. Indeed, in terms of construct validity, these authors (e.g., Anthony et al., 2022a) found that data observed using the SSIS SEL brief scales–student form supported a two-factor factorial solution: the social awareness factor and a second factor consisting of the remaining factors collapsed into a single latent dimension. This study, by providing evidence of high overlap among certain CASEL competency domains, underscored the need to carefully examine and evaluate the factor structure implied by the CASEL framework in diverse cultural contexts, including the Italian sample.

Additionally, Anthony et al. (2023) further verified the questionnaire’s factorial invariance across different age-based cohorts of students, recommending a future investigation of the instrument’s psychometric properties as a function of “other characteristics as well” (p.15). The study offers key evidence on the factorial structure of the SSIS SEL brief scales–student form measurement model. However, certain questions remain to be answered. Could the “collapsed factor” be viewed as a second-order factor in the Italian cultural context? Does the proposed model remain invariant across gender-based groups of students? Is it possible to test a specifically Italian measurement model that could help to address the challenges (e.g., a high correlation between factors) posed by previous measurement models?

Given this background, the current article presents the analytical approach we used to validate the Italian version of the SSIS SEL brief scales–student form. We begin by examining how SEL was previously measured in assessment research (from the CASEL model onwards), moving on to discuss the SSIS SEL brief scales and how this instrument performed in measurement approaches in the past. We then describe the present study’s participant sample, data collection procedures, and data analysis strategy. Finally, we explore the potential for deploying the SSIS SEL brief scales in the field and offer suggestions for its future use in both research and educational contexts.

## 2. What is social and emotional learning?

SEL is defined as the process through which children and adolescents acquire and apply the knowledge, skills, and attitudes they need to develop healthy identities, manage their emotions, achieve personal and collective goals, feel and show empathy for others, establish and maintain supportive relationships, and make responsible decisions (Durlak et al., 2015). According to the CASEL model, SEL is composed of five interrelated core competencies, namely self-awareness, self-management, social awareness, relationship skills, and responsible decision-making (Mahoney et al., 2021). Self-awareness skills are defined as the ability to accurately recognize one’s emotions and thoughts and their influence on one’s behaviors. This includes knowing how to accurately assess one’s own strengths and limitations. Self-management skills are defined as the ability to effectively regulate one’s emotions, thoughts, and behaviors across different situations. This involves managing stress, controlling one’s impulses, motivating oneself, and working to achieve personal, academic, and collective goals. Social awareness skills imply the capacity to adopt the perspective of others from diverse backgrounds and cultures, to display empathy, compassion, and gratitude, to understand social and ethical behavioral norms, and to recognize the resources and forms of support available within one’s family, school, and community. Relationship skills are defined as the ability to establish and maintain healthy and rewarding relationships with others. This involves communicating clearly, actively listening, cooperating, negotiating conflict constructively, and seeking and offering help as appropriate. Finally, responsible decision-making skills imply the ability to make constructive and respectful choices that foster personal, social, and collective well-being, in keeping with ethical standards, safety considerations, and social norms (Mahoney et al., 2021).

Various comprehensive, universal, and multi-year SEL programs have been widely implemented in the United States over the past three decades. Recent studies, including meta-analyses, have shown that evidence-based SEL programs have a significant impact on the five earlier-listed dimensions of SEL over both the short and the long term (e.g., Cefai and Cavioni, 2015; Murano et al., 2020). More specifically, such meta-analyses documented significant gains in school achievement (Corcoran et al., 2018; Blewitt et al., 2021), self-management and relational skills (Boncu et al., 2017), and prosocial attitudes (Durlak et al., 2011; Luo et al., 2022). They also show that promoting SEL significantly reduces both internalizing problems, such as anxiety and depression, and externalizing behaviors, such as violence and high-risk conduct (Goldberg et al., 2019; Cavioni et al., 2020a; Durlak et al., 2022).

Although comprehensive SEL programs have recently been developed to promote SEL in schools across Europe also (see, for instance, Agliati et al., 2020; Berg et al., 2021; Cefai et al., 2022), there is currently a lack of validated measures for evaluating SEL in European countries (Anthony et al., 2022a). A recent review (Cavioni and Grazzani, 2023) identified one exception to this pattern: the Devereux Student Strengths Assessment (DESSA) battery, which was first standardized in the United States (LeBuffe et al., 2014) and then adapted for use in Italy. It includes scales that assess social and emotional learning in students aged 5 to 13 years and is available in teacher and parent forms but lacks a student form.

Translated versions of the SSIS SEL brief scales recently were administered within a large European study that examined multiple indicators of mental health including resilience and SEL. The Scales, which are available in parent, teacher, and student forms, measure the SEL skills of children aged 3–18 and yield highly reliable evidence. In the earlier-cited study by Anthony et al. (2023), cross-country measurement invariance was tested with data from Croatia, Greece, Italy, Latvia, Portugal, and Romania. The authors found a high degree of measurement invariance across countries, supporting the use of translated versions of the SSIS SEL brief scales in international research programs.

## 3. The present study

The aim of this study was to further contribute to refining the measurement model underpinning the SSIS SEL brief scales–student form by exploring alternative solutions such as a unidimensional and second-order hierarchical model. To this end, we analyzed data gathered from a large sample of Italian children and adolescents, conducting additional exploratory and confirmatory factor analyses with a view to identifying a model that offers a better fit for the Italian setting. Once a valid factor solution had been identified, as a second aim we took the resulting measurement model as our baseline and used it to test for invariance between boys and girls. Indeed, in the context of refining and validating a measurement model for the SSIS SEL brief scales–student form, evidence relating to gender invariance will crucially inform the real-world application of the instrument.

## 4. Method

### 4.1. Participants

The sample consisted of 1,175 children and adolescents attending primary, lower secondary, and upper secondary schools in Northern Italy. All participants were taking part in the large-scale PROMEHS universal intervention study which involved the design and testing of a school-based curriculum for the promotion of mental health that included a focus on enhancing SEL (e.g., Cefai et al., 2022; Colomeischi et al., 2022; Cavioni et al., 2023). However, for the purpose of this research, only the pre-test data were utilized. The participating students’ mean age was 11.02 years (SD = 2.42; age range = 8–16 years) and 46.8% (*n* = 550) were boys. The distribution of participants by age was as follows: 8–9 years (*n* = 442, 37.6%), 10–11 years (*n* = 280, 23.8%), 12–13 years (*n* = 190, 16.2%), 14–16 years (*n* = 263, 22.4%). The inclusion criteria for the study were as follows: (1) being between the ages of 8 and 16 years at the time of the study, (2) being enrolled at an Italian school, and (3) having agreed to the terms of participation in the research. We applied no exclusion criteria. We recruited a convenience sample using a non-probability sampling technique (Emerson, 2015). The data were collected anonymously from students to protect their privacy and ensure confidentiality. Participants were not required to provide their names or surnames on the questionnaires; instead, an alphanumeric code was assigned to each student.

All participants were made aware of the objectives and procedures of the study. The Ethics Board of Milano-Bicocca University (Protocol number: 0044281/20 obtained on the 21st/7/ July 2020) approved the research, which was conducted in accordance with the ethical principles outlined in the Declaration of Helsinki (World Medical Association, 2013) and the American Psychological Association code of conduct (Knapp and VandeCreek, 2004).

### 4.2. Measure and procedures

The SSIS brief scales series (SSIS SEL brief scales–student form; Elliott et al., 2020) is a multi-informant assessment of 3- to 18-year-old students’ SEL competencies. The series includes also scales for teachers, who can complete the instruments both for themselves and for their students (more detailed information is provided in Elliott et al., 2020). According to Anthony et al. (2022b), the original sample utilized for the validation of the SSIS SEL brief scale-student form consisted of 530 student forms collected from two Midwestern private/parochial schools. One of the schools exhibited a diverse distribution of students across various grades, encompassing a comprehensive age range within the sample. Among the participants in this school, 45% were identified as female, and 55% were identified as male, indicating a balanced gender representation. Most students from this school were White (93.5%), with other racial backgrounds comprising a smaller percentage of African American (0.4%), Asian (1.3%), Hispanic (1.7%), and multiracial (3%). Participants from the second private school (*n* = 179) were roughly evenly distributed across kindergarten through 4th grade and were evenly distributed across sex, with 50% being boys and 50% being girls. Further demographic data for this school were not available, but the demographics indicate that approximately 70% of students were White, and around 30% receive needs-based tuition assistance.

We conducted a validation study of the score inferences from the SSIS SEL brief scales–student form for students aged 8–18 years, which comprises 20 items designed to match the CASEL framework of SEL competencies. Each of the five SEL domains is assessed on a four-point scale from 0 (Not True) to 3 (Very True). The student is presented with a list of items (e.g., Item 2: “I stay calm when dealing with problems”; Item 12: “I pay attention when the teacher talks to the class”) and decides how true each sentence is for him/her (not true; a little true; a lot true; very true).

In line with International Testing Commission guidelines (International Test Commission, 2017), the Italian translation of the scale (see Appendix 1; to download the tool see also: https://www.labpse.it/strumenti) was developed through a forward and backward translation procedure and with the input of a native English speaker who is also fluent in Italian.

### 4.3. Data analysis

**Figure 1:**
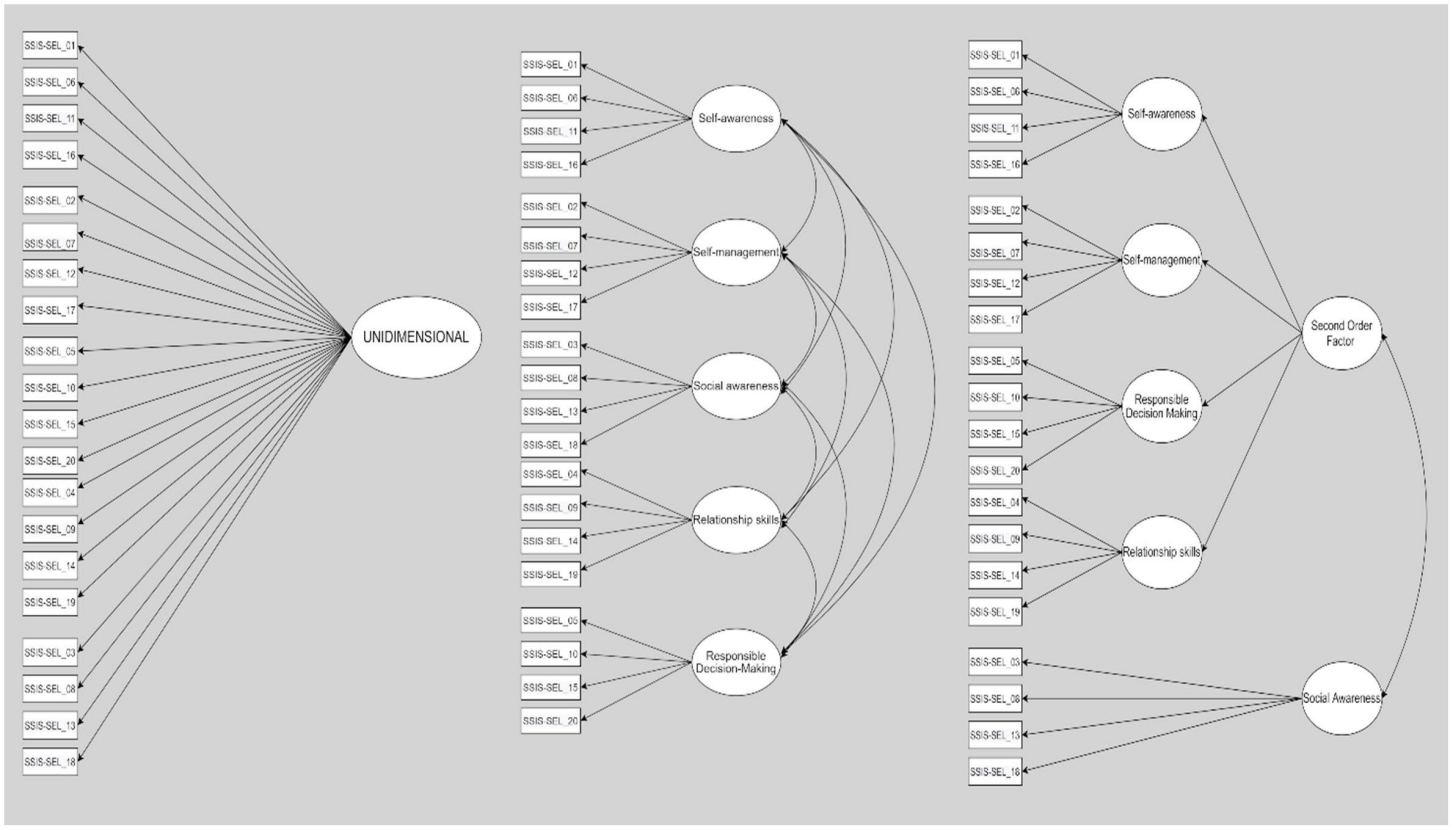
Confirmatory factor analysis outcomes for the Italian dataset: representation of a four-factor model resembling the original factor structure of the social skills improvement system social and emotional learning (SSIS SEL) brief scales–student form. *All values were statistically significant at *p* < 0.001.

The data analysis strategy comprised two stages. At the first stage, three different measurement models were tested. The first was a unidimensional model with all items loading on a single latent factor (Model A). The second was the five-factor model (Model B) originally suggested for the SSIS SEL brief scales–student form, while the third (Model C) was a second-order hierarchical measurement model [as hypothesized by Anthony et al. (2023)]. The last-mentioned model predicted the existence of a second-order factor (generating the dimensions of social awareness, relationship skills, self-management, and responsible decision-making), and social awareness as a separate first-order factor (see Figure 1).

Figure 1 summarizes the three conceptual models initially tested. On the left is the unidimensional measurement model (Model A) with all items loading onto a single latent factor. The original measurement model for the SSIS SEL brief scales–student form (Model B) is illustrated at the center of the figure. A second-order measurement model (Model C), with social awareness as a distinct first-order factor and all other dimensions loading onto a higher-order factor, is shown on the right.

In seeking to adapt the measurement model of the SSIS SEL brief scales–student form to our target population, we followed multivariate confirmatory factor analysis (CFA) procedures (Kline, 2013; Cavalera et al., 2017; Grazzani et al., 2017; Conte et al., 2019). Confirmatory factor analysis provides empirical and numerical support for the development of quantitative instruments by testing how well a given measurement model fits a set of empirical data. First, however, we assessed the data for the normality of distribution. None of the items obtained kurtosis or skewness values that fell outside the recommended ranges of 2 to 4 for the former criterion and − 1 to 1 for the latter criterion (Blanca et al., 2013). We then checked the data for multivariate outliers by calculating Mahalanobis distances (Ghorbani, 2019). Finally, we adopted the Maximum Likelihood method to estimate the Structural Equation Model (Ripplinger and Sullivan, 2008).

To estimate model fit, we calculated five goodness-of-fit indices: the Root Mean Square Error of Approximation (RMSEA), the Standardized Root Mean Square Residual (SRMR), the Normed Fit Index (NFI), the Tucker-Lewis Index (TLI, TLI >0.95), and the Comparative Fit Index. We viewed a model as fitting the data if the RMSEA was under 0.07 (Kenny et al., 2015), the SRMR under 0.05 (Ximénez et al., 2022), and the NFI over 0.95 (Shi and Maydeu-Olivares, 2020). We estimated confidence limits with a set of 200 random samples, in accordance with current recommendations for SEM (Thakkar, 2020). In addition, we adopted the Akaike information criterion (AIC) to check the general fit of the models and compare them to one another.

Finally, we used multi-group CFA (MGCFA; Byrne, 1998) to test the invariance of the best-fitting structural model across gender-based groups. The hypothesis of group invariance was to be accepted if configural invariance (i.e., the underlying relationships between variables are stable across groups), metric invariance (i.e., the model parameters, such as regression coefficients, bear the same meaning across groups), and scalar invariance (i.e., the unit of measurement is the same across groups) were all supported (see Van De Schoot et al., 2015). Equivalence of the model across groups was to be rejected if the difference between the target model and the nested models was statistically significant. We set the cutoff criteria for rejecting invariance at Δ > 0.01 for both ΔRMSEA and ΔSRMR (Chen, 2007) and a chi-square difference (Δχ^2^) that was statistically significant at the *p* < 0.01 level (Milfont and Fischer, 2010). The different types of invariances are hierarchically ordered, meaning that the MGCFA procedure ends at the lowest level of invariance that fails to be satisfied (for further details, see Cheung and Rensvold, 2002).

At the second stage of the data analysis, exploratory and confirmatory factor analysis were applied to further refine the measurement model underlying the SSIS SEL brief scales. Given the ordinal nature of the item scores (which were based on a Likert response scale), Explanatory Factor Analysis (EFA) was applied to a polychoric correlation matrix computed using Lorenzo-Seva and Ferrando (2015) syntax for SPSS (Holgado-Tello et al., 2010). We also performed the Kaiser Meyer-Olkin (KMO) and Bartlett’s sphericity tests (Field, 2009) to assess the suitability of the data for factor analysis. We drew on Kaiser’s criterion (K1; Kaiser, 1960) to determine the most appropriate number of factors to retain and parallel analysis (Horn, 1965) to help us identify the best factor structure for the adapted Italian version of the questionnaire. Parallel analysis (PA) is a data simulation technique that compares the eigenvalues of a set of observed data with those of randomly generated data sets of comparable size (Hayton et al., 2004). Ultimately, we only included factor loadings (λ) of over 0.50 (Hair et al., 2006), discarding items that loaded on multiple factors (Costello and Osborne, 2005). The EFA yielded a “baseline” factor structure which we could then further evaluate by using CFA and MGCFA to test measurement invariance across gender-based subgroups (the statistical criteria adopted were the same as those applied during the first stage of the data analysis to test the fit of the three models).

We used the software applications Statistical Package for the Social Sciences 25.0 (SPSS; Pituch and Stevens, 2015) and Analysis of Moment Structures 25.0 (AMOS; Arbuckle, 2011) for all the analyses. Missing values (at item level) were replaced at random following the range of the response scale. Missing valuer represented less than 1% of available data.

## 5. Results

The results section is divided into two parts that reflect the aims of the study. In the first section, we report the results of the confirmatory factor analyses performed on the three initially hypothesized measurement models. The second section presents the measurement model validated for the Italian setting based on exploratory factor analysis, confirmatory factor analysis, and an analysis of measurement invariance across male and female cohorts.

### 5.1. Confirmatory factor analysis on three alternative measurement models for the SISS SEL brief scales–student form

Table 1 summarizes the descriptive analysis of the data.

**Table 1:** Descriptive statistics: SSIS SEL brief scales–student form (*N* = 1,175).

	min	max	*M*	*SD*
SSIS-SEL_01	1	4	2.68	0.849
SSIS-SEL_02	1	4	2.22	0.874
SSIS-SEL_03	1	4	3.33	0.745
SSIS-SEL_04	1	4	2.99	0.837
SSIS-SEL_05	1	4	2.46	0.819
SSIS-SEL_06	1	4	3.14	0.791
SSIS-SEL_07	1	4	2.38	0.932
SSIS-SEL_08	1	4	3.19	0.811
SSIS-SEL_09	1	4	3.29	0.809
SSIS-SEL_10	1	4	3.44	0.745
SSIS-SEL_11	1	4	2.72	0.908
SSIS-SEL_12	1	4	2.98	0.794
SSIS-SEL_13	1	4	3.17	0.807
SSIS-SEL_14	1	4	3.65	0.608
SSIS-SEL_15	1	4	3.12	0.781
SSIS-SEL_16	1	4	3.10	0.740
SSIS-SEL_17	1	4	2.77	0.899
SSIS-SEL_18	1	4	3.03	0.845
SSIS-SEL_19	1	4	3.11	0.831
SSIS-SEL_20	1	4	3.31	0.761

Next, the unidimensional model was estimated. The model converged and the minimum was achieved, but the absolute and relative goodness of fit indexes did not support a measurement model composed of 20 empirical indicators loading on a single dimension: χ^2^(170) = 1,133,99, *p* < 0.001, NC = 6.67, NFI = 0.775, TLI =0.778, CFI = 0.802, RMSEA = 0.069, 90% CI [0.065–0.073], SRMR = 0.056, AIC = 1,253.99.

Next, the fit of the original five-factor measurement model was assessed and this time the fit indexes were more robust χ^2^(153) = 400.34, *p* < 0.001, NC = 2.61, NFI = 0.921, TLI =0.937, CFI = 0.949, RMSEA = 0.037, 90% CI [0.032–0.041], SRMR = 0.035, AIC = 554.34. Model fit values were calculated by specifying six error covariances. A closer inspection of the model’s parameters brought two critical issues to light. First, the covariance matrix was not positive definite. Secondly, high inter-factor covariances suggested significant overlap among factors. More specifically, the standardized covariance values were 0.95 for Self-Management (F2) and Responsible Decision-Making (F5), 0.95 for Relationship Skills (F4) and Self-Awareness (F1), and 0.86 for Self-Awareness (F1) and Responsible Decision-Making (F5). Furthermore, a standardized value of greater than one (1.02) was obtained for Self-Awareness (F1) and Self-Management (F2). Taken as a whole, these critical issues with the original five-factor model (as already pointed out by Anthony et al., 2023) prompt non-acceptance.

Lastly, the second-order measurement model offered a good fit for the data: χ^2^(157) = 399.56 *p* < 0.001, NC = 2.54, NFI = 0.921, TLI =0.940, CFI = 0.950, RMSEA = 0.036, 90% CI [0.032–0.040], SRMR = 0.035, AIC = 545.56. Model fit values were calculated by specifying eight error covariances. Although the fit indexes were relatively good, the model was non recursive and therefore could not be accepted. In addition, two negative covariances were estimated in relation to F1 (−0.005) and F2 (−0.009). Overall, the results did not support a second-order measurement model for the SSIS SEL brief scales–student form. In general, the results of this first stage in our data analysis did not lead to a definitive conclusion as to which measurement model should be used to interpret SSIS SEL brief scales–student form scores. Consequently, we conducted a fresh exploratory factor analysis in order to uncover patterns of association that were more specific to the Italian cultural context.

### 5.2. Exploratory and confirmatory factor analysis and analysis of measurement invariance for the SISS SEL brief scales–student form in the Italian context

EFA was applied to the scores of a randomly selected half-sample (training set), and subsequently, CFA was applied to the scores of the remaining half-sample (testing set). The Kaiser-Meyer-Olkin (0.89) test and Bartlett’s test of sphericity (2,585, *p* < 0.001) confirmed that the data were suitable for factor analysis. The outcomes of the EFA suggested that four distinct factors explained 46% of the variance. Parallel analysis also supported a four-factor solution (see Table 2).

**Table 2:** Exploratory factor analysis outcomes for the Italian dataset (N = 598).

	1	2	3	4
SSIS-SEL_10	0.670			
SSIS-SEL_20	0.661			
SSIS-SEL_12	0.639			
SSIS-SEL_16	*0.485*			
SSIS-SEL_14	*0.484*			
SSIS-SEL_15	*0.461*			
SSIS-SEL_08		0.802		
SSIS-SEL_18		0.744		
SSIS-SEL_03		0.699		
SSIS-SEL_13		*0*.695		
SSIS-SEL_17		*0.414*		
SSIS-SEL_19		*–*		
SSIS-SEL_11			0.721	
SSIS-SEL_01			0.719	
SSIS-SEL_09			0.503	
SSIS-SEL_07				0.729
SSIS-SEL_02				0.643
SSIS-SEL_05				0.507
SSIS-SEL_04				*0.433*
Eigenvalue	5.29	1.69	1.15	1.07
Variance	26.46	8.49	5.75	5.36
Cumulative variance	26.46	34.95	40.71	46.07

Loading values of under 0.500 are reported in italics.

Seven items from the original pool failed to satisfy the criteria for acceptance (i.e., λ > 0.50) and were therefore excluded from the measurement model. The first factor was measured by items related to responsible decision-making and the second factor by items measuring social awareness. The third factor expressed self-management skills while the fourth factor reflected self-awareness. The fifth factor in the original model (relationship awareness) was not identified by the EFA and was therefore omitted from the subsequent analyses. The resulting factor structure partially overlapped with that originally identified for the SSIS SEL brief scales–student form. The new measurement model (four factors, 13 items) was adopted as the baseline structure for further confirmatory factor analysis and multigroup invariance analysis.

**Figure 2:**
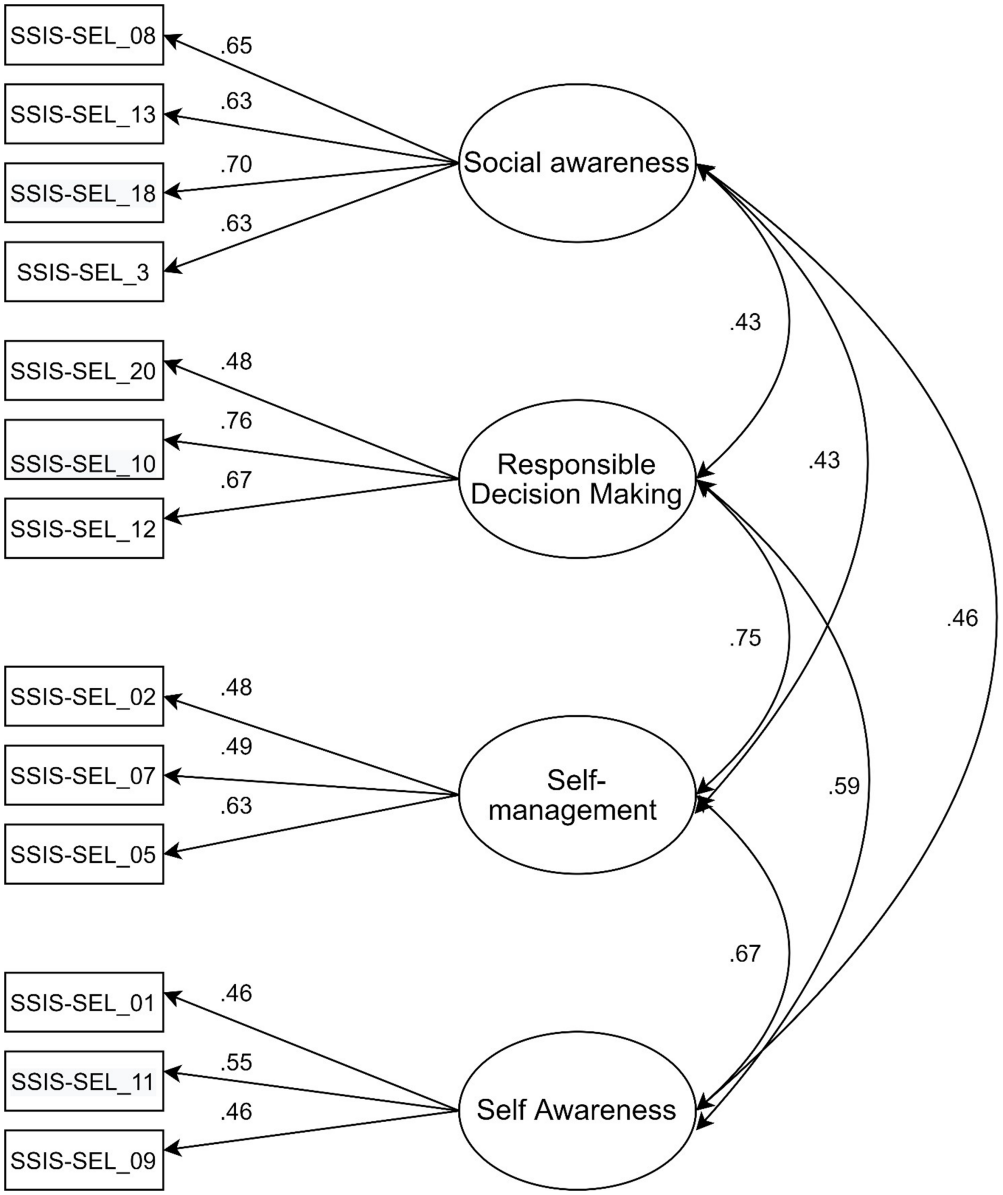
The three conceptual models.

The baseline measurement model offered an excellent fit with the Italian dataset: χ^2^(58) = 92.56 *p* = 0.003, NC = 1.59, NFI = 0.939, TLI =0.968, CFI = 0.976, RMSEA = 0.032, 90% CI [0.019–0.043], SRMR = 0.033, AIC = 184.6. In addition, the minimum was achieved, the model was recursive, and no negative variances were found. This result was attained by constraining only one co-variance, and specifically between the error terms of Items 10 and 20. Figure 2 presents the standardized direct path coefficients and the standardized correlations between the factors.

Table 3 summarizes the outcomes of the multigroup invariance test of the model with four correlated factors and 13 items. The MGCFA partially supported the hypothesis of invariance across gender-based groups. Specifically, two levels of invariance were confirmed by robust statistical values in terms of absolute fit as well as a comparison between models. None of the other nested models (e.g., structural covariance) met the criteria for acceptance, meaning that the SSIS SEL brief scales–student form was not found to display any of the more complex types of measurement invariance. Overall, the results indicated partial measurement invariance, implying that equivalence of item intercepts between gender-based groups could not be assumed or used to draw conclusions (Clark and Donnellan, 2021).

**Table 3:** Outcomes of the structural model comparison between gender-based cohorts.

Model	χ^2^ (df)	CFI	RMSEA	[90% CI]	Model comparison	Δχ^2^ (Δdf)	ΔCFI	ΔRMSEA	Decision
Ma. Configural invariance	197.2 (116)	0.970	0.024	0.018–0.030	–	–	–	–	Accept
Mb. Metric invariance	216.6 (125)	0.966	0.025	0.019–0.030	Ma	19.4 (9)	0.004	0.001	Accept
Mc. Scalar invariance	344.0 (137)	0.924	0.030	0.031–0.041	Mb	127.4 (12)	0.042	0.005	Reject
Md. Full invariance	373.4 (148)	0.917	0.036	0.032–0.040	Mc	29.4 (11)	0.010	0.006	Reject

## 6. Discussion

The aim of this study was to validate the score inferences from the Italian version of the SSIS SEL brief scale student form by testing a range of factor structures in search of a theoretically and statistically sound factor solution for the Italian sample. Given that SEL involves culturally situated skills and behaviors (Conte et al., 2018; Allbright et al., 2019; Cahill and Dadvand, 2020; Hayashi et al., 2022), well-designed assessment tools must be aligned with models of children’s social and emotional competencies within specific cultural settings.

The outcomes of the multiple confirmatory and exploratory analyses that we conducted on the Italian dataset were consistent with those of other studies (Anthony et al., 2022a, 2023), suggesting the need for caution in adopting measurement models for the SSIS SEL brief scale student form. Indeed, none of the three models initially tested (unidimensional, originally validated, and second-order) offered a completely satisfactory fit for the Italian data. This prompted us to apply exploratory factor analysis in search of a new and more suitable measurement model. The baseline structural model with four factors and 13 items identified via the EFA was supported by both subsequent confirmatory factor analysis and the measurement invariance test. The factor structure of the Italian version of the SSIS SEL brief scale–student form partially overlaps with that of the original measurement model: it retains four of the five original factors while dropping the dimension of relationship skills. These four factors, and their relative items, were essentially confirmed by our analysis, with only minor differences emerging regarding the allocation of three specific items. The factorial structure and configuration of the various items as observed in the Italian context are discussed below.

### 6.1. Italian version of the SISS SEL brief scale–student form: a closer look at its key dimensions

The dimension of *self-awareness*, namely understanding one’s own emotions, thoughts, and values and how they influence behavior in different contexts, was expressed by two CASEL-aligned items, including the item “I look at people when I talk to them” (λ = 0.51), which in the original model was classified as a component of relationship awareness. Plausibly, in the Italian cultural context and the age group under study, looking another person in the face is a behavior that may be driven by self-confidence rather than by relationship awareness. Looking at another person, as Garland-Thomson (2009) reminds us in his book Staring: How We Look, is a culturally determined behavior, whose meanings can vary considerably as a function of context.

The *social awareness* domain concerns the ability to understand and empathize with the perspectives of others, including those from diverse backgrounds, cultures, and contexts. It includes the ability to feel compassion for others, comprehend broader social and ethical norms for behavior in various settings, and identify the resources and support structures available within one’s family, school, and community. Interestingly, according to Anthony et al. (2023), the items representing this latent factor remained identical to the original set aligned with the CASEL model, being perfectly expressed in the Italian dataset.

*Self-Management* is the ability to effectively manage one’s emotions, thoughts, and behaviors in a variety of situations to achieve one’s goals and aspirations. This includes the ability to delay gratification, manage stress, feel motivated, and have the agency to achieve personal/collective goals. Our factor analysis supported the retention of two original items and the addition of the item “I do the right thing without being told” (λ = 0.51). In this case, it arguably makes sense to include “doing the right thing” under the umbrella of self-management rather than under the dimension of responsible decision-making (as in the original model). Indeed, throughout their lives, children and young people are frequently urged to “do the right thing” by various social actors, including parents, relatives, teachers, and (especially) peers. It is worth noting that such behaviors are philosophically ambiguous, in the sense that “moral worth is a positive status that some, but not all, morally right actions possess” (Johnson King, 2020). In other words, for a child or adolescent, acting appropriately in a given life context may often require more self-management skills than responsible decision-making.

The *Responsible Decision Making* dimension entails the ability to make caring and constructive decisions concerning one’s personal behavior and social interactions across a variety of situations. This includes the ability to take ethical standards and safety concerns into account, as well as to assess the benefits and consequences of various actions on personal, social, and collective well-being. Our new structural model confirmed two of the original items for this dimension, adding the item “I pay attention when the teacher talks to the class” (=0.65), which was previously attributed to the dimension of self-management. The inclusion of the new item makes numerical and theoretical sense, insofar as listening to teachers is more likely to be driven by a general sense of conscientiousness (thereby drawing on cognitive and emotional domains) rather than by self-management ability, which appears to be less salient to the school setting.

With regard to the second aim of the study, the four-factor measurement model displayed partial invariance across male and female cohorts in that only metric invariance was supported by the data.

The observation of partial gender invariance raises critical concerns about the performance of the SISS SEL brief scales-student form across genders, indicating that the instrument might not measure the same underlying construct in boys and girls with complete equivalence. The identification of such partial measurement invariance means that specific items on the scale evoke distinct response patterns in male and female individuals. As a result, direct comparisons of scores on these specific items between genders may not adequately reflect true gender differences in Social and Emotional Learning (SEL) experiences. The result prompts us to critically evaluate whether the observed differences between genders are genuine reflections of SEL disparities or if they are influenced by the variations in item responses due to gender-specific factors. By addressing these challenges, we can improve the validity and dependability of our findings while also contributing to the advancement of gender-sensitive research in quantitative psychology, enabling more accurate and comprehensive insights into the dynamics of Social and Emotional Learning (Grazzani et al., 2020; Cavioni et al., 2020b).

Although the upholding of metric invariance suggests that discrepancies across gender groups were not generated by the measuring scale *per se*, differences between groups may arise for other reasons. For instance, gender roles and societal expectations may differ between male and female participants, leading to variations in how they perceive and respond to SEL items (Fabes and Martin, 1991; Fischer, 2000; Shields, 2003). Several studies observed the presence of gender-related differences in various social and emotional competencies. As a way of example, Zhao et al. (2014) found significant variations between boys and girls in their approach to emotion regulation. Other studies also indicated that women may be more emotionally responsive than men (Fujita et al., 1991; Bradley et al., 2001). However, as suggested by McRae et al. (2008), one limitation of studies based on self-report methods is their vulnerability to the effects of gender stereotypes because they ask participants to report their experiences retrospectively. Such stereotypes, related to the expression and understanding of emotions as well as in language use and communication styles in emotion-related topics can shape individuals’ self-reporting and may contribute to the observed partial invariance. While these studies did not directly focus on social and emotional learning using the CASEL model (Mahoney et al., 2021), their findings highlight how gender roles and societal expectations can influence several aspects of the emotional and social domains. Once these biases are carefully accounted for and minimized, the reported gender differences in emotion-related outcomes tend to diminish or even disappear (Barrett et al., 1998; Gard and Kring, 2007). Future researchers are encouraged to continue to explore the construct validity of the assessment instrument to explore the underlying reasons behind partial invariance, seeking to untangle the intricate relationships between gender, cultural factors, and the measurement of SEL. Metric invariance analyses may not be sufficient to confirm the reliability and validity of outcomes if the variables evaluated by the instrument are significantly influenced by gender differences. In sum, interpreting partial invariance between males and females will require both a detailed examination of the measurement model’s strengths and weaknesses and a fuller understanding of the gender differences under study.

### 6.2. On the adoption of the SISS SEL brief scales–student form in research and intervention processes

The present study, building upon the previous work by Anthony et al. (2023), aimed at contributing to the growing body of research on SEL by examining the factor structure of the Italian version of the SSIS SEL brief scales-student form. Exploring the empirical fit of the framework’s constructs in the Italian sample, we aimed at elucidating the relevance and applicability of the CASEL model to a diverse cultural setting, enriching our understanding of SEL constructs and their measurement across different cultural contexts.

Given that a four-factor solution offered the best match for the self-rating of SISS SEL items collected from a large sample of Italian children and adolescents, we believe it is reasonable to maintain this factor structure in future SISS SEL studies in the Italian context, acknowledging its cultural specificity (Miller-Cotto et al., 2022). Therefore, we would especially recommend adopting the four-factor structure in studies aimed at advancing theoretical understanding of SEL, given the stronger statistical profile obtained for this measurement model.

One significant finding that requires attention is the non-inclusion of seven items out of the original 20 in the final measurement model. It is plausible that differences in cultural norms or conceptual interpretations between the original US/English version and the translated Italian version may contribute to the observed discrepancies. These discrepancies highlight the importance of cultural adaptation and localization when utilizing measurement instruments in different cultural settings. Indeed, SEL can be influenced by values, norms, and educational practices, which may differ between the United States and Italy.

While on numerical and methodological grounds we identified the four-factor model as the best fitting solution for SISS SEL, it is important to note that the initial CFA did not suggest complete rejection of a two-dimensional factor solution, an outcome that is in line with the findings of previous studies (Anthony et al., 2023). We, therefore, believe that the solution with two latent dimensions should be considered when practitioners use the SISS-SEL for comparative purposes, given that only this model of measurement has been tested across multiple European contexts to date (*ibidem*). In other words, collapsing the factor structure may be more appropriate in comparative studies. Finally, while a robust factor structure and internal consistency are key indicators of trustworthy measures, it is vital to recognize that in applied educational settings (especially when children and adolescents are involved), researchers need brief screening instruments or abbreviated versions of existing SEL evaluation tools. A robust (but lengthy) paper-and-pencil questionnaire with several dozen items measuring a range of components and requiring a long time to complete may be administered in conventional research settings. However, researchers should bear in mind that poor functioning (e.g., high missing response rates, the tendency to provide standard responses, and sample selection bias) can impact the accuracy of ordinarily successful (but lengthy) screening tools in everyday contexts. “The shorter, the better” is a good rule of thumb to follow when conducting field research. This also applies to screening instruments. Hence, switching from a 20-item version of the SSIS SEL to a 13-item version could be beneficial in some instances.

### 6.3. Limitations and future research

Four limitations of this study should be noted. Firstly, while the study aimed to gather a large sample from northern Italy, the participants may not fully statistically represent the entire Italian student population. This limitation can affect the generalizability of the results to all Italian students. Secondly, focusing the analysis on this sample may restrict the extent to which the results can be extrapolated to other contexts. Hence, cultural and social factors could influence how students perceive and respond to SEL items, warranting caution when interpreting the findings beyond the Italian setting. Thirdly, the use of self-report questionnaires relies on participants’ subjective responses, which may be influenced by social desirability bias. This could impact the reliability of the data collected. Finally, the results of the Italian validation of the SSIS SEL brief scales-student form demonstrated a four-factor structure, which deviated from the original instrument’s five-factor structure. This discrepancy could suggest potential differences in how the instrument measures SEL in the Italian student population compared to the original sample. While this four-factor structure identified in our sample offers valuable insights into the SEL dimensions among Italian students, it may represent a limitation in terms of direct comparison with results from other countries that have previously validated or applied the original five-factor structure (Anthony et al., 2022a).

## 7. Conclusion

Increasing interest in SEL programs on the part of practitioners, researchers, and policy-makers in Europe as well as in the United States is driving the need for efficient and validated instruments for assessing SEL competencies across different countries, age groups and genders. In this study, we have contributed to the validation of the Italian version of the SSIS SEL brief scales for male and female students aged between 8 and 16 years. This represents the first stage in the country-level validation of the Italian-adapted versions of the SSIS SEL brief scales. The following steps will involve validating the SSIS SEL teacher and parent scales, using the data collected during the implementation of the PROMEHS Mental Health Promotion Project.

## Data availability statement

The raw data supporting the conclusions of this article will be made available by the authors, without undue reservation.

## Ethics statement

The studies involving humans were approved by the Ethics Committee of the University of Milano-Bicocca (protocol code: 0044281/20). The studies were conducted in accordance with the local legislation and institutional requirements. Written parental consent consent for participation in this study was provided by the participants’ legal guardians/next of kin.

## Author contributions

VC, EC, IG, VO, and AP coordinated the development and writing of the manuscript, and made key contributions to designing the research, interpreting the data, and drafting and revising the manuscript. CC made a substantial contribution to the conception and design of the research, and the revision of the manuscript. VC and EC contributed to data collection. VO and AP made a relevant contribution to the data analysis. CA and SE made significant contributions to interpreting the data and revising the manuscript. All authors contributed to the article and approved the submitted version.

## Figures and Tables

**Table:** 

	Non vero	Un po’ vero	Molto vero	Del tutto vero
1. Chiedo aiuto quando ne ho bisogno (SV)				
2. Rimango calmo/a quando affronto problemi (SV)				
3. Aiuto i miei amici quando hanno dei problemi (SV)				
4. Lavoro bene con i miei compagni e compagne di classe				
5. Faccio la cosa giusta senza che me lo si dica(SV)				
6. In gruppo, faccio la mia parte				
7. Resto calmo/a quando sono in disaccordo con gli altri (SV)				
8. Difendo gli altri quando non vengono trattati bene (SV)				
9. Guardo le persone quando parlo con loro (SV)				
10. Sto attento/a quando uso cose che non sono mie (SV)				
11. Faccio sapere alle persone quando c’è un problema (SV)				
12. Sto attento/a quando l’insegnante parla alla classe (SV)				
13. Cerco di far stare meglio gli altri (SV)				
14. Dico “grazie” quando qualcuno mi aiuta				
15. Mantengo le promesse				
16. Sto attento/a quando gli altri espongono le loro idee				
17. Cerco di trovare un buon modo per porre fine a un disaccordo				
18. Cerco di capire come si sentono gli altri (SV)				
19. Cerco di perdonare gli altri quando dicono “scusa”				
20. Rispetto le regole scolastiche (SV)				

## Footnotes

^1^ www.promehs.org
